# ki67 nuclei detection and ki67-index estimation: a novel automatic approach based on human vision modeling

**DOI:** 10.1186/s12859-019-3285-4

**Published:** 2019-12-27

**Authors:** Barbara Rita Barricelli, Elena Casiraghi, Jessica Gliozzo, Veronica Huber, Biagio Eugenio Leone, Alessandro Rizzi, Barbara Vergani

**Affiliations:** 1 Department of Information Engineering, Università degli Studi di Brescia, Via Branze 38, 25123 Brescia, Italy; 2 Department of Computer Science, Università degli Studi di Milano, Via Celoria 18, 20133 Milan, Italy; 3 Fondazione IRCCS Ca’ Granda - Ospedale Maggiore Policlinico, Department of Dermatology, Viale Regina Marghertita, 20122 Milan, Italy; 4 Unit of Immunotherapy of Human Tumors, Department of Research, Fondazione IRCCS Istituto Nazionale dei Tumori, Milan, Italy; 5 School of Medicine and Surgery, Università degli Studi di Milano-Bicocca, Via Cadore 48, 20900 Monza, Italy

**Keywords:** Color enhancement, Human vision model, Image processing, Image segmentation, Artificial intelligence, Histochemical image analysis, ki67 cell nuclei counting

## Abstract

**Background:**

The protein ki67 (pki67) is a marker of tumor aggressiveness, and its expression has been proven to be useful in the prognostic and predictive evaluation of several types of tumors. To numerically quantify the pki67 presence in cancerous tissue areas, pathologists generally analyze histochemical images to count the number of tumor nuclei marked for pki67. This allows estimating the ki67-index, that is the percentage of tumor nuclei positive for pki67 over all the tumor nuclei. Given the high image resolution and dimensions, its estimation by expert clinicians is particularly laborious and time consuming. Though automatic cell counting techniques have been presented so far, the problem is still open.

**Results:**

In this paper we present a novel automatic approach for the estimations of the ki67-index. The method starts by exploiting the STRESS algorithm to produce a color enhanced image where all pixels belonging to nuclei are easily identified by thresholding, and then separated into positive (i.e. pixels belonging to nuclei marked for pki67) and negative by a binary classification tree. Next, positive and negative nuclei pixels are processed separately by two multiscale procedures identifying isolated nuclei and separating adjoining nuclei. The multiscale procedures exploit two Bayesian classification trees to recognize positive and negative nuclei-shaped regions.

**Conclusions:**

The evaluation of the computed results, both through experts’ visual assessments and through the comparison of the computed indexes with those of experts, proved that the prototype is promising, so that experts believe in its potential as a tool to be exploited in the clinical practice as a valid aid for clinicians estimating the ki67-index. The MATLAB source code is open source for research purposes.

## Background

In the anatomopathological field, experts often concentrate on the visual analysis of histochemical images; indeed, immunohistochemistry allows producing high-resolution images where proteins of interest are visualized through specific stains by exploiting the principle of (labelled) antibodies binding specifically to antigens in biological tissues. Particularly, the expression of the human ki67 protein (pki67) is strictly associated with cell proliferation [1–5].

Being associated with the proliferative activity of cell populations, the pki67 is nowadays used as a marker of tumor aggressiveness [6, 7], and several research studies have already investigated the utility of its quantification in the prognostic and predictive evaluation of several types of tumors, such as breast, meningioma, soft tissue, lung, prostate, cervix and central nervous system cancers [8–17] and [18–21].

The expression of pki67 in cancerous tissue areas is quantified by the so-called ki67-index, that is the percentage of tumor nuclei positive for pki67 (positive nuclei) over all the tumor nuclei (positive nuclei and negative nuclei). Clinical experts estimate it in a visual way by counting positive and negative nuclei through a careful observation of histochemical images where cells are marked through apposite colorants. Though nuclei counting protocols have been defined [22] to help obtaining precise counting results, researches have shown that the ki67-index is one of the hardest to compute [23, 24]. Indeed, visual inspection is a laborious and time-consuming task that produces not-replicable and not accurate estimates, affected by high inter- and intra-observer variability [25–27]; this failure is probably due to the huge dimension of the treated tissue images that cannot be exhaustively analyzed by experts, due to their limited time. As a result, in the clinical routine, the ki67-index is never measured by applying state of the art counting procedures to precisely count nuclei [22], but it is visually estimated by observing the expression of pki67 in a limited number of small tissue patches, and averaging the results.

Thanks to the advent, and subsequent proliferation, of whole-slide digital scanners, together with the continuous increase in computational power, and the substantial advances in the digital image processing and pattern recognition fields, in the past decade a lot of clinical and research work has been devoted to the development of Computer Aided Diagnosis (CAD) systems [28–30] helping pathologists during their analysis of immunohistochemical (IHC) images [31]. Reviews such as those presented in [32–41] are evidence of the continuous and increasing interest in the development of CAD analyzing histological images to identify nuclei. Moreover, studies specifically focusing on the segmentation of pki67 and on the estimation of the ki67 labeling index [42–46] highlight the clinical need of an automated system providing an accurate, repeatable, and trustable estimate. Unfortunately, despite the major research effort targeted and focused on ki67 expression analysis from histological images, the problem is still widely open.

Indeed, image problems, depending on the presence of tissue folds and/or cuts, unspecific colorations, uneven color cast, and unwanted background structures, misguide the image analysis systems [47, 48]. Though some promising methods exist, the quality of their results often depends on several thresholds whose tuning is particularly difficult for users such as clinicians, who are not computer science experts. Other methods use particularly complex image processing techniques, and given the high image resolutions and dimensions, they are too expensive in terms of computational time and memory storage. Finally, in the latest years, some effective deep learning methods have been presented [43, 49] that could solve this problem thanks to their impressive generalization capability; however, due to the high number of parameters to be learnt even when using transfer learning [50], they require a huge training set obtained by manual labeling procedures. As an example, the interesting work proposed in [43], identifies isolated nuclei thanks to a deep network, which is trained on a labeled set composed of 450 microscopic images with 2048 × 1536 pixel. The images have been extracted from 90 (histologically confirmed) slides, and contain almost 259,884 nuclei (131,053 immunopositive and 128,831 immunonegative) nuclei. To estimate the time needed to create such a huge training set, we asked three experts, E5, E15 and E30, with respectively five, fifteen and thirty years of expertise in the field, to label ten image patches with dimension of 1024 × 1024 pixels, and to record the time spent while labeling. To speed the manual labeling process, the three experts alternately worked at the labeling. It took 50 h (5 h per image patch) to label the ten patches. Due to clinicians’ work overload, spending so much time for manual training is not acceptable, and hampers the ample application of these effective state-of-the-art deep learning methods. Moreover, learning techniques, and especially deep learning techniques, are black-boxes which are often avoided in the clinical field when “interpretations” are at the basis of research. Though the problem of “interpretable” machine learning techniques has recently started to be seriously investigated in literature [51–56], this research field is still at its early stage and lacks well established techniques for providing either interpretations of predicted output, or counterfactual explanation, which explain how to modify the input to obtain a different output. The lack of interpretations cause clinicians to mistrust machine learning techniques and deep learning techniques and prefer rule-based systems.

Despite the aforementioned difficulties, automatic analysis is increasingly demanded for its objective, precise and repeatable numerical estimates on a statistically significant number of high-resolution images.

In this paper, we present our prototype solution to the problem of automatically estimating the ki67-index. To augment the visibility of marked (positive) and unmarked (negative) nuclei, our method firstly applies the Spatio-Temporal Retinex inspired Envelope with Stochastic Sampling (STRESS) algorithm [57], a “Spatial Color Algorithm” [58] (SCA) that enhances colors, increases contrast and compensates for color cast. As a result, nuclei whose brightness could cause under-segmentation are made evident. Furthermore, when nuclei clusters are present, contrast enhancement has the effect of increasing color difference among adjacent nuclei; in this way, separation of adjoining nuclei is made easier. After this preprocessing stage, a simple thresholding step allows us to segment pixels belonging to all the nuclei, both those positive and those negative for pki67 (positive nuclei and negative nuclei, respectively). This step is followed by a Bayesian tree classifier, which is an interpretable machine learning techniques whose rules allow identifying marked and unmarked pixels based on three color features. Each of the two binary masks (positive nuclei mask and negative nuclei mask) identifying, respectively, marked and unmarked pixels contains both isolated regions, corresponding to isolated nuclei, which can be recognized for they have an “acceptable” area and a round shape, and bigger regions with an inadequate area and shape, which correspond to nuclei clusters. Since positive and negative nuclei differ for their morphological shape, to identify single nuclei in the two masks, they are processed separately. At first, to recognize regions with a round shape similar to nuclei, two Bayesian trees employing morphological features have been trained. One Bayesian tree recognizes eligible positive nuclei shapes. The other Bayesian tree recognizes eligible negative nuclei shapes. The Bayesian trees are then used to classify the regions selected by two consecutive multiscale procedures, applied separately to the positive nuclei mask and to the negative nuclei mask. The first method employs Laplacian of Gaussian filters (at different scales), while the second method applies a modified version of the STRESS algorithm (with different radii). The proposed method effectively identifies both isolated regions and nuclei belonging to nuclei clusters. It has been evaluated by counting nuclei on 105 sections or fields acquired with different resolutions and settings, and then comparing the computed estimates to those obtained by three experts; the promising results computed by the presented approach confirm its potential as a valid tool to be used in the clinical routine basis as an aid to pathologists.

This paper is structured as described in the following. Section 2 describes the results achieved by the research study. Precisely, in subsection 2 the developed method for the automatic count of cell nuclei is presented; in section 2.3 we report experiments performed to test the robustness of our method with respect to different image acquisitions, and different parameter settings, in subsection 5.1 we describe the images used for developing and testing the presented work. Finally, conclusions and future works are reported in section 3.

## Results

In this section, we describe the result of our research work, which is a prototype for the estimation of the ki-67 index. In particular, after enhancing the image colors (see subsection 2.4), a classifiers (see subsection 2.2) is used to extract markers characterized by any color and shape; secondly, two consecutive multiscale approaches (see subsection 2.5 and subsection 2.6) process the segmented areas to detach clustered nuclei and detect eligible nuclei shapes thanks to a second classifier (see subsection 2.3). The experimental results (see subsection 2.7) show the effectiveness of our method.

### Learning the color appearance of nuclei-pixels and the morphological appearance of nuclei

In this section, we describe the classification trees used in the following steps of our method.

The first Bayesian tree, referred as ${BT}_{Color}^{3 Class}$ in the following, employs color features to classify pixels as belonging to either background, positive, or negative nuclei, while the two other Bayesian trees, referred as ${BT}_{Shape}^{POS}$ and ${BT}_{Shape}^{NEG}$ in the following, are used to select binary regions whose shape is similar to that of positive or negative nuclei respectively. To let clinicians select training pixels and shapes, we have developed a simple user interface that shows sample sub-images and asks experts to draw polygons around positive nuclei, negative nuclei, and background regions.

### Training of ${BT}_{Color}^{3 Class}$

The manual labeling procedure identifies *NPos* + *Nneg* + *Nback* pixels that are separated into the three classes containing, respectively, all pixels in positive nuclei regions, all pixels in negative nuclei regions, all pixels in background regions. Each pixel is characterized by a color *p*_*color*_ expressed either in the RGB color space, that is *p*_*color*_ = {*R*_*p*_, *G*_*p*_, *B*_*p*_}, or in the HSV color space, that is *p*_*color*_ = {*H*_*p*_, *S*_*p*_, *V*_*p*_}. Coding each pixel *p* as a 3D vector *p*_*coded*_ = {*R*_*p*_, *B*_*p*_, *H*_*p*_}, whose features are the red- and blue-channel color values from the RGB representation and the hue value from the HSV color representation, a training set composed of coded pixels and their labels (*POS*, *NEG*, *BACK*) is formed and used as input to train a Bayesian tree classifier, which classifies each coded pixel as belonging to one of the following three classes: background pixel, positive nuclei pixel (positive pixels), negative nuclei pixel (negative pixels).

### Training of ${BT}_{Shape}^{POS}$ and ${BT}_{Shape}^{NEG}$

To capture the information about the nuclei shape, from the manually drawn positive/negative and background regions, we have first computed the minimum area among all positive (*minAP*) and all negative regions (*minAN*), the two median areas (*medAP*, *medAN*), the two maximum areas (*maxAP*, *maxAN*), and the minimum (*minRP*, *minRN*), the median (*medRP*, *medRN*), and the maximum (*maxRP*, *maxRN*) among the radii of the positive and the negative nuclei regions.

Next, each manually labelled nuclei region has been coded by computing morphological properties such as: the compactness (*Comp*), the eccentricity (*Ecc*), the length of the minor (*MinAxis*) and major (*MaxAxis*) axis of the ellipse containing the region, the area of the convex hull (*Aconvex*), the perimeter (*P*), the area (*A*), the ratio of area and perimeter $\left(\frac{P}{A}\right)$, the minimum (*minRad*) and maximum (*maxRad*) distance among the area border and the area skeleton, the ratio $\frac{minRad}{maxRad}$, the bounding box of the region (*BB*), the ratio $\left(\frac{A}{BB}\right)$, and the ratios $\left(\frac{A}{minAP},\frac{A}{medAP},\frac{A}{maxAP},\frac{MinAxis}{minRP},\frac{MaxAxis}{maxRP},\frac{MinAxis}{medRP},\frac{MaxAxis}{medRP}\ \right)$ for positive areas, while the ratios $\left(\frac{A}{minAN},\frac{A}{medAN},\frac{A}{maxAN},\frac{MinAxis}{minRN},\frac{MaxAxis}{maxRN},\frac{MinAxis}{medRN},\frac{MaxAxis}{medRN}\ \right)$ for negative regions.

Briefly, each positive region has been represented by a vector of 20 features:

1 $$ {\mathrm{Reg}}_{\mathrm{P}\mathrm{os}}=\left[\mathrm{Comp},\mathrm{Ecc},\mathrm{MinAxis},\mathrm{MaxAxis},\mathrm{A}\mathrm{convex},\mathrm{P},\mathrm{A},\frac{\mathrm{P}}{\mathrm{A}},\mathrm{minRad},\mathrm{maxRad},\frac{\mathrm{minRad}}{\mathrm{maxRad}},\mathrm{BB},\frac{\mathrm{A}}{\mathrm{BB}},\kern0.5em \frac{\mathrm{A}}{\mathrm{minAP}},\frac{\mathrm{A}}{\mathrm{medAP}},\frac{\mathrm{A}}{\mathrm{maxAP}},\frac{\mathrm{MinAxis}}{\mathrm{minRP}},\frac{\mathrm{MaxAxis}}{\mathrm{maxRP}},\frac{\mathrm{MinAxis}}{\mathrm{medRP}},\frac{\mathrm{MaxAxis}}{\mathrm{medRP}}\ \right] $$

Similarly, each negative region has been represented by a vector of 20 features:

2 $$ {\mathrm{Reg}}_{\mathrm{Neg}}=\left[\mathrm{Comp},\mathrm{Ecc},\mathrm{MinAxis},\mathrm{MaxAxis},\mathrm{A}\mathrm{convex},\mathrm{P},\mathrm{A},\frac{\mathrm{P}}{\mathrm{A}},\mathrm{minRad},\mathrm{maxRad},\frac{\mathrm{minRad}}{\mathrm{maxRad}},\mathrm{BB},\frac{\mathrm{A}}{\mathrm{BB}},\kern0.5em \frac{\mathrm{A}}{\mathrm{minAN}},\frac{\mathrm{A}}{\mathrm{medAN}},\frac{\mathrm{A}}{\mathrm{maxAN}},\frac{\mathrm{MinAxis}}{\mathrm{minRN}},\frac{\mathrm{MaxAxis}}{\mathrm{maxRN}},\frac{\mathrm{MinAxis}}{\mathrm{medRN}},\frac{\mathrm{MaxAxis}}{\mathrm{medRN}}\right] $$

Regarding background areas, they have been coded twice to relate the background regions to both the positive and the negative nuclei regions. The first coding comprises the features:

3 $$ {\mathrm{Reg}}_{\mathrm{BACK}}^{\mathrm{P}\mathrm{os}}=\left[\mathrm{Comp},\mathrm{Ecc},\mathrm{MinAxis},\mathrm{MaxAxis},\mathrm{A}\mathrm{convex},\mathrm{P},\mathrm{A},\frac{\mathrm{P}}{\mathrm{A}},\mathrm{minRad},\mathrm{maxRad},\frac{\mathrm{minRad}}{\mathrm{maxRad}},\mathrm{BB},\frac{\mathrm{A}}{\mathrm{BB}},\kern0.5em \frac{\mathrm{A}}{\mathrm{minAP}},\frac{\mathrm{A}}{\mathrm{medAP}},\frac{\mathrm{A}}{\mathrm{maxAP}},\frac{\mathrm{MinAxis}}{\mathrm{minRP}},\frac{\mathrm{MaxAxis}}{\mathrm{maxRP}},\frac{\mathrm{MinAxis}}{\mathrm{medRP}},\frac{\mathrm{MaxAxis}}{\mathrm{medRP}}\right] $$

while the second coding comprises the features:

4 $$ {\mathrm{Reg}}_{\mathrm{BACK}}^{\mathrm{NEG}}=\left[\mathrm{Comp},\mathrm{Ecc},\mathrm{MinAxis},\mathrm{MaxAxis},\mathrm{A}\mathrm{convex},\mathrm{P},\mathrm{A},\frac{\mathrm{P}}{\mathrm{A}},\mathrm{minRad},\mathrm{maxRad},\frac{\mathrm{minRad}}{\mathrm{maxRad}},\mathrm{BB},\frac{\mathrm{A}}{\mathrm{BB}},\kern0.5em \frac{\mathrm{A}}{\mathrm{minAN}},\frac{\mathrm{A}}{\mathrm{medAN}},\frac{\mathrm{A}}{\mathrm{maxAN}},\frac{\mathrm{MinAxis}}{\mathrm{minRN}},\frac{\mathrm{MaxAxis}}{\mathrm{maxRN}},\frac{\mathrm{MinAxis}}{\mathrm{medRN}},\frac{\mathrm{MaxAxis}}{\mathrm{medRN}}\right] $$

Note that the only difference in the coding of the background areas is in the last seven features, which relate the morphological description of the region to the statistics collected by the manual segmentation.

The coded regions have been used to form two training sets. The first training set has been used to train the Bayesian tree, ${BT}_{Shape}^{POS}$, recognizing shapes similar to those of positive nuclei. It is composed by *NReg*_*POS*_ vectors coding the manually drawn *NReg*_*POS*_ positive nuclei regions (Reg_POS_(*i*) for all *i* = 1, …, *NReg*_*POS*_) plus *NReg*_*BACK*_ vectors coding the manually drawn *NReg*_*BACK*_ background regions (${\mathrm{Reg}}_{\mathrm{BACK}}^{\mathrm{Pos}}(i)$ for all *i* = 1, …, *NReg*_*BACK*_). Note that, in this case, the coding vector is the one that relates background regions to positive nuclei regions (see Eq. 3).

Similarly, the second training set has been used to train the Bayesian tree, ${BT}_{Shape}^{NEG}$, recognizing shapes similar to those of negative nuclei. It is composed by *NReg*_*NEG*_ vectors coding the manually drawn *NReg*_*NEG*_ negative nuclei regions (Reg_Neg_(*i*) for all *i* = 1, …, *NReg*_*NEG*_) plus *NReg*_*BACK*_ vectors coding the manually drawn *NReg*_*BACK*_ background regions (${\mathrm{Reg}}_{\mathrm{BACK}}^{\mathrm{NEG}}(i)$ for all *i* = 1, …, *NReg*_*BACK*_). Note that, in this case, the coding vector is the one that relates background regions to negative nuclei regions (see Eq. 4).

The described classification trees are used by the prototype as described in what follows.

### Image enhancement and rough nuclei segmentation

**Fig. 1:**
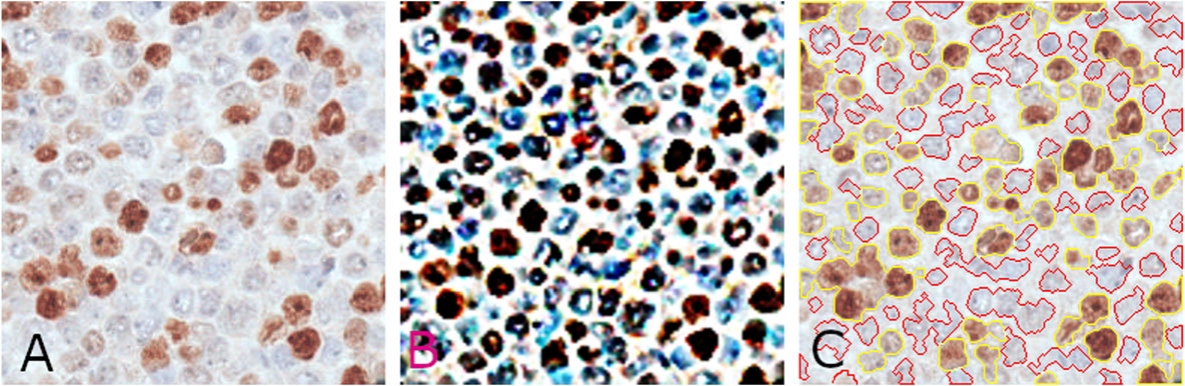
Results of the rough segmentation step. Left (**a**): original sub-image. Center (**b**): color-enhanced image after applying STRESS. Right (**c**): the borders of segmented nuclei areas are highlighted in yellow (nuclei positive for pki67) and red (nuclei negative for pki67). Lots of clustered nuclei are visible

The first step of the prototype detects all the pixels belonging to nuclei that are both positive and negative for pki67. This step must overcome difficulties due to low contrasted nuclei characterized by a feeble color, which are considered by experts as subtle for they are “barely visible”. Besides, some nuclei are often “weakly positive” for pki67 and are therefore characterized by a color appearance that is a mixture of brownish and blueish. As an example, in the sub-image in Fig. 1a the reader may observe that some blue nuclei are characterized by a light color sometimes very similar to the background; furthermore, some positive nuclei have a low contrasted bluish appearance. To obtain an effective segmentation we firstly enhance color and contrast in the processed images by applying the STRESS algorithm [57], a color compensation algorithm which has shown to provide effective results when applied for image dehazing [59], enhancing astrophotographs images [60], and spatio-temporal color correction of movies [61].

STRESS is a spatial color algorithm, where each pixel *p*_0_ is assigned a new RGB value computed as the mean of *N* stretched color (RGB) values. Each stretched color value is computed by stretching the value of *p*_0_ between the minimum and maximum color values among those obtained by sampling *M* pixels in a circular neighborhood of radius *R* around *p*_0_. STRESS is therefore governed by parameters *N* (number of iterations), *M* (number of sampled value), and *R* (the radius of the sampling area centered on each pixel *p*_0_ to be modified. When treating 20x (40x) images, we set them to *N*_20*x*_ = 128, *M*_20*x*_ = 8 (*N*_40*x*_ = 256, *M*_40*x*_ = 16), though other values have proven to produce similar results (as shown in the experimental results, Section 2.7). Regarding parameter *R*, its value is set to be the length of the maximum radius of the nuclei to be detected, that is *R* =  *max* (*maxRP*, *maxRN*). An example of image resulting from the application of STRESS is shown in Fig. 1b. The algorithm produces impressive results; in the resulting RGB color image, “barely visible” nuclei are brought out and made clearly visible, as per opinion of experts E5, E15 and E30**.**

After applying STRESS, candidate nuclei pixels are simply detected by selecting all the pixels for which the mean value over the red, green, and blue color channels is less than or equal to 225 (this threshold value has been experimentally set, though we experimented also values in the range [200,…, 240], which produce similar results).

**Fig. 2:**
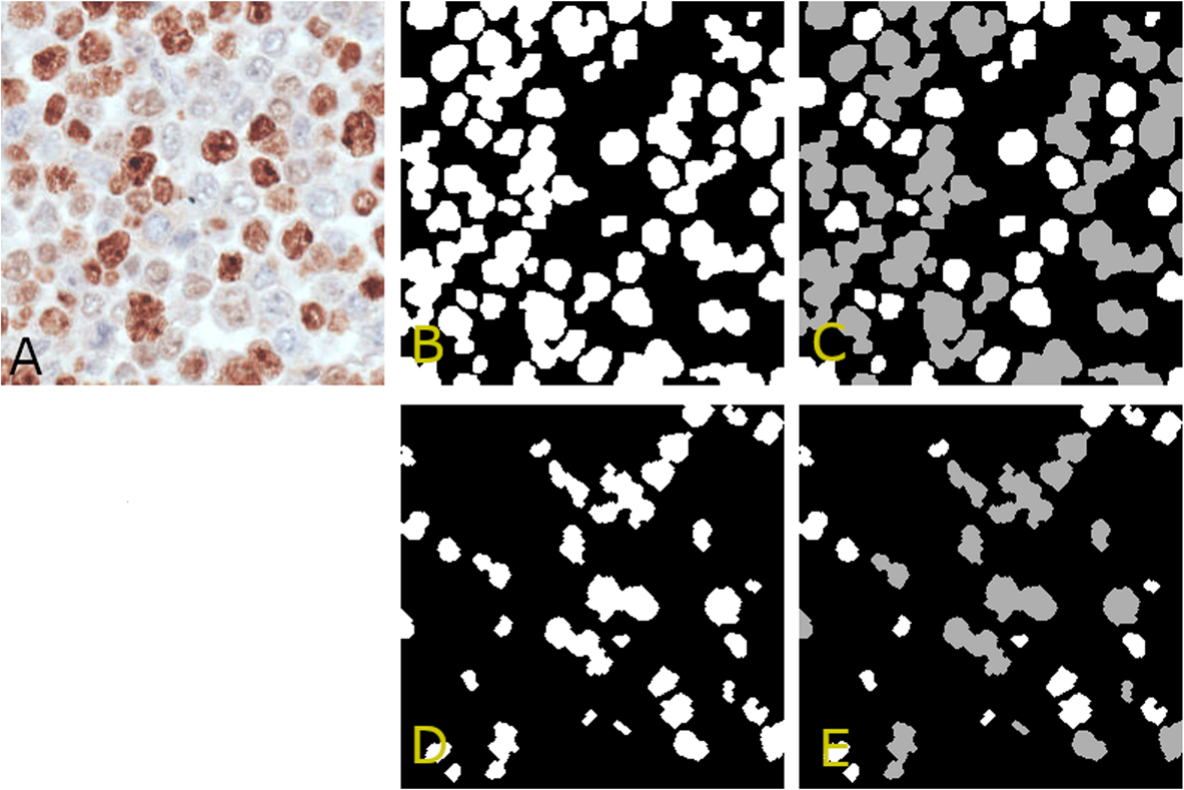
Nuclei masks. **a**: sample sub-image. **b**: positive nuclei mask identifying pixels belonging to positive nuclei. **c**: round shaped regions (white) and regions left in the positive nuclei mask (gray). **d**: negative nuclei mask identifying pixels belonging to negative nuclei. **e**: round shaped regions (white) and regions left in the negative nuclei mask (gray)

The color of the selected candidate pixels are then coded as described in subsection 2.1.1 and fed as input to ${BT}_{Color}^{3 Class}$ with the aim of discarding false positive pixels, and separate pixels belonging to positive nuclei from those belonging to negative nuclei. In this way, false positive pixels belonging to background are discarded, while the remaining pixels are split into two binary masks, called ${m}_{nuclei}^{POS}$ and ${m}_{nuclei}^{NEG}$ in the following, that identify, respectively, pixels belonging to positive nuclei and pixels belonging to negative nuclei (see Figs. 1c and 2b, d).

Figure 1 shows a sample sub-image on the left (A), the image resulting from the application of the STRESS algorithm (B), and the classification result (C), which has been achieved by training ${BT}_{Color}^{3 Class}$ with pixels contained in 30 background areas (for a total of 3477 pixels), 34 negative nuclei with a median area of about 115 pixels (for a total of 3904 negative pixels), and 37 positive nuclei with median area of about 192 pixels (for a total of 7056 positive pixels) from two sub-images (note that in our image database positive nuclei are generally bigger than negative ones). In Fig. 1c the borders of the computed binary nuclei-masks (which will be simply referred as ${m}_{nuclei}^{POS}$, for positive nuclei, and ${m}_{nuclei}^{NEG}$, for negative nuclei, in the following) are shown; precisely, the borders of ${m}_{nuclei}^{POS}$ are yellow, while the borders of ${m}_{nuclei}^{NEG}$ are red. The reader may observe that in both masks, nuclei are connected, and lots of nuclei clusters are present in the segmentation results. In the next subsections, we describe a multiscale refinement procedure, which is aimed at analyzing the pixels in the computed masks to identify and differentiate clustered nuclei from isolated ones.

Regardless, both E15 and E30 assessed the segmentation and classification results computed by this segmentation step and issued a favorable judgment. Precisely, no false positive area was found to be included into the segmented areas, while few nuclei areas remained undetected; however, both E15 and E30 considered them as negligible for they are barely visible.

### Multiscale nuclei detection by Laplacian of Gaussian (log) filtering

The prototype must analyze the pixels identified by the nuclei masks (see Fig. 2b, d) to detect circular regions of varying radii. For this reason, we employ a multiscale approach and apply it separately on the pixels in ${m}_{nuclei}^{POS}$ and ${m}_{nuclei}^{NEG}$. In the following, we describe the procedure we applied to the pixels identified by the generic mask, referred to as ${m}_{nuclei}^{\ast }$. Note that the described procedures employs ${BT}_{Shape}^{POS}$ (see subsection 2.1.2) when working on ${m}_{nuclei}^{POS}$, and ${BT}_{Shape}^{NEG}$ when working on ${m}_{nuclei}^{NEG}$. In the following the employed Bayesian tree will be referred and ${BT}_{Shape}^{\ast }$.

Precisely, given the computed nuclei mask ${m}_{nuclei}^{\ast }$, the first step applies ${BT}_{Shape}^{\ast }$ to detect isolated nuclei shaped regions (see subsection 2.1.2); the detected regions are recorded in the final result and removed from ${m}_{nuclei}^{\ast }$ to avoid considering them in the following step (see Fig. 2c, e). Next, the multiscale approach is applied on the gray level sub-image *I*_*gray*_.

Specifically, to detect blob-like structures, *I*_*gray*_ is filtered with Laplacian of Gaussian filters [62] with varying radii and standard deviations. Each filter has a radius value *r* in the range [*rMin*, *rMax*], which are respectively the minimum and the maximum of all the radii of the manually signed nuclei regions; the standard deviation of the LoG filter with radius *r* equals $\frac{1}{3}r$.

**Fig. 3:**
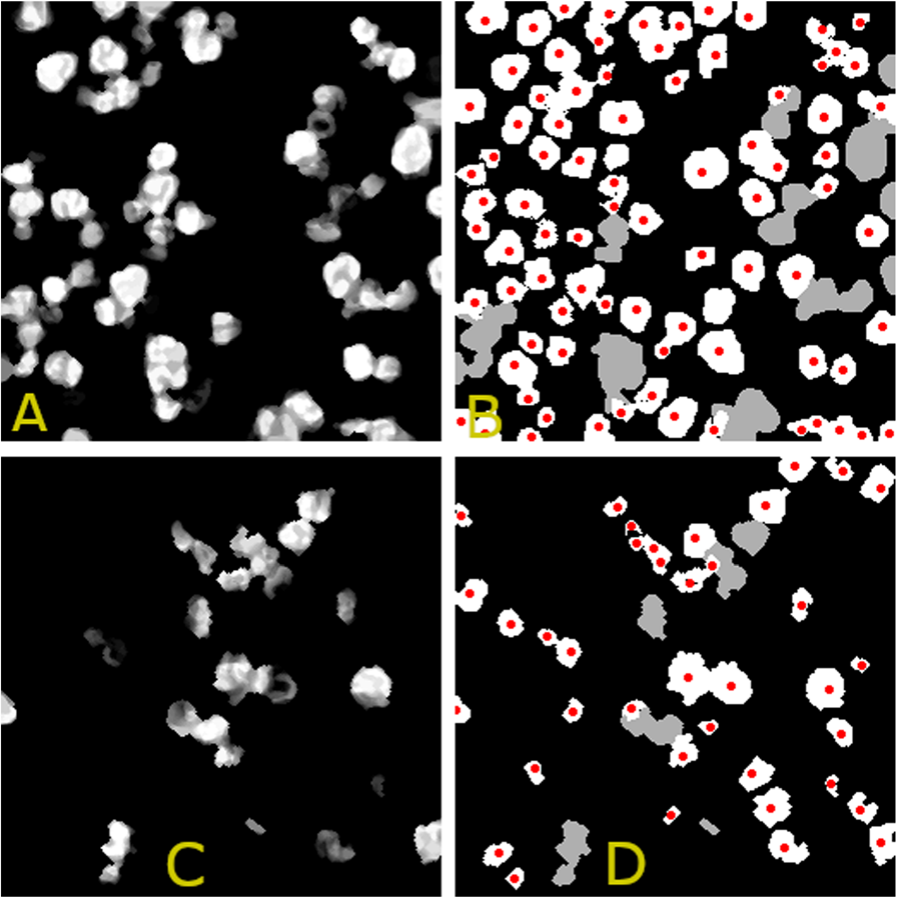
Voting images and result of the multiscale approach. Top (**a**, **b**): analysis of the positive nuclei mask. Bottom (**c**, **d**): analysis of the negative nuclei mask. Left (**a**, **c**): voting image. Right (**b**, **d**): the detected nuclei (white) are identified by their centroid (red). The gray areas are those left in the nuclei mask

After each filtering, the filtered image *Ilog*_*r*_ is thresholded by keeping the 65% of the pixels in $$ {m}_{nuclei}^{\ast } $$ with the highest value (the percentage value of 65% has been experimentally chosen, though values in the range [55%, ...,75%] are also well suited). When the filtering iteration ends, each pixel in $$ {m}_{nuclei}^{\ast } $$ has a vote that tells how many times the pixels has been selected by the thresholding procedure. All votes are recorded in an image $$ {I}_{VOTES}^{\ast } $$, where only pixels in the mask can take a value different from zero. For the sake of clarity, Fig. 3 shows the voting images $$ {I}_{VOTES}^{POS} $$ and $$ {I}_{VOTES}^{NEG}, $$ obtained for the positive (Fig. 3a) and the negative nuclei (Fig. 3c). It may be noted that in the voting images, $$ {I}_{VOTES}^{\ast }, $$ clustered nuclei are visible. To separate them, we iteratively threshold the voting image. Precisely, for each connected region in $$ {m}_{nuclei}^{\ast } $$, we keep a percentage, *perc*_*Log*_ (*perc*_*Log*_ ∈ {75, 60, 45, 30, 15}), of pixels with the highest value in $$ {I}_{VOTES}^{\ast } $$. After each thresholding, the connected regions formed by the selected pixels are fed to $$ {BT}_{Shape}^{\ast } $$ to detect eligible nuclei regions. The detected regions are recorded in the final results and removed from $$ {m}_{nuclei}^{\ast } $$.

In the right column of Fig. 3 we show with white color the positive (Fig. 3b) and negative (Fig. 3d) nuclei regions (with their centroids in red) detected by the described multiscale processing. Gray colored regions are those that are still left in the mask ${m}_{nuclei}^{\ast }$**.**

### Multiscale nuclei detection by stress filtering

Regions that are still present in ${m}_{nuclei}^{\ast }$ after the multiscale procedure described above are often characterized by low contrast, so that the separation among adjacent nuclei is barely visible. To enhance the color contrast in those regions we have filtered *I*_*gray*_ with a modified version of STRESS (referred as “masked STRESS” in the following), which differs from STRESS because it employs a mask to filter the randomly chosen samples around the point *p*_0_. Precisely, for each iteration, masked STRESS randomly samples *M* points among those located in a binary mask and laying within a distance *R* from *p*_0_. Using a mask to restrict the allowable samples, masked STRESS is obliged to work on the range of colors covered by pixels in the mask, thus creating major contrast where needed.

**Fig. 4:**
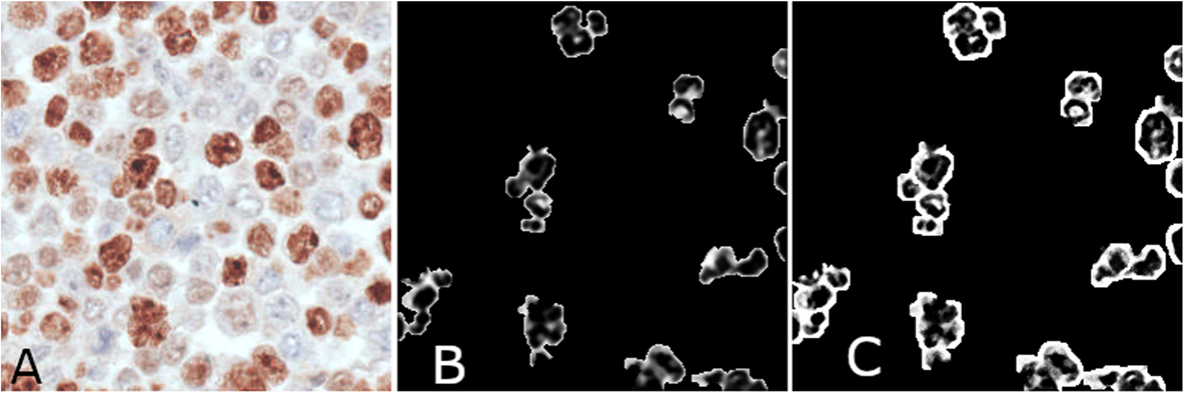
Comparison of results obtained by STRESS (**b**) and masked STRESS (**c**). Both the images have been computed by using parameters R = 14, N = 128, M = 8

To allow a visual comparison, in Fig. 4 we show the results computed by applying STRESS (Fig. 4b) on *I*_*gray*_ (with parameter values *R* = 14, *N* = 128, *M* = 8) and those computed by applying masked STRESS (Fig. 4c) with the same parameter values. In the picture produced by masked STRESS, the separation among nuclei is more evident.

To detect and separate nuclei in the regions that are left in the mask ${m}_{nuclei}^{\ast },$ we consider each region separately. Precisely, given the binary region *reg* contained in ${m}_{nuclei}^{\ast }$, we start computing the median and the minimum of all the region thicknesses, [*thick*_*min*_, …, *thick*_*med*_] (the minimum and the median thicknesses of a binary region *reg* are computed as the minimum and the median of all the distances among the skeleton of *reg* and the points on the perimeter of *reg*). Next for each thickness value, *thick* ∈ [*thick*_*min*_, …, *thick*_*med*_], we apply to *I*_*gray*_ by the masked STRESS algorithm with parameter values *N* = 128, *M* = 8, *R* = *thick*, and using *reg* as the mask. Next, the resulting image is processed by employing an iterative thresholding procedure similar to that described in subsection 2.5. Precisely, at each iteration, we select a percentage, *perc*_*STRESS*_ (*perc*_*STRESS*_ ∈ {85, 70, 55, 40, 25 }), of the pixels with the lowest value; connected regions in the obtained binary image are then analyzed to detect eligible nuclei regions. Precisely, we select as nuclei regions those connected regions characterized by all the following properties:

- the area is less than the maximum eligible area (that is *maxAP* for positive nuclei, and *maxAN* for negative nuclei, see subsection 2.1)
- the area is bigger than half of the smallest eligible area (that is *minAP* for positive nuclei, and *minAN* for negative nuclei, see subsection 2.1),
- the ratio among the length of the minor (*min*_*Axis*_) and the major (*max*_*Axis*_) axis of the ellipse containing the region is $\frac{{\mathit{\min}}_{Axis}}{{\mathit{\max}}_{Axis}}>0.6$.

Selected regions are then removed from *reg* and recorded in the final result. This iterative procedure is repeated for the values of the aforementioned values of *perc* or until *reg* is empty.

When all the regions have been processed with the aforementioned iterative procedure, the mask ${m}_{nuclei}^{\ast }$ generally contains only small regions generally corresponding to nuclei whose shape is very different from those seen by the Bayesian tree classifiers. These regions are anyway added to the final result.

**Fig. 5:**
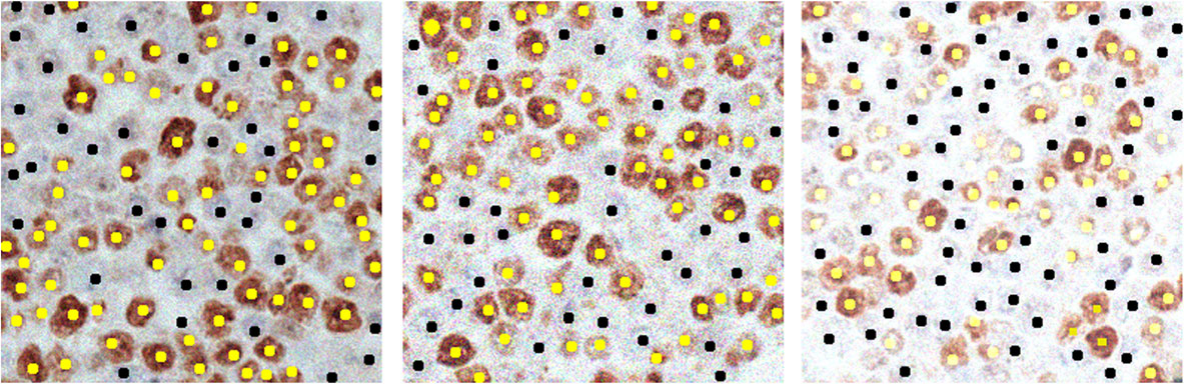
Example of computed results. The centroids of detected nuclei are marked in yellow for positive nuclei, and in dark blue for negative nuclei

To allow a visual assessment of the achieved results, in Fig. 5 we show 4 sub-images where the centroids of the detected nuclei have been superimposed in yellow (positive nuclei) and in black (negative nuclei). Results are promising though the images are quite noisy and characterized by different color characteristics.

We recall that, as described in section 5.1.1, each sub-image belong to a manually identified tumor region, and that each tumor region is characterized by its own proliferation activity, described by the ki67-index estimate for that region. To estimate the ki67-index for a specific tumor region, T, when all the nuclei (positive for ki67 and negative for ki67) are found in all the sub-images belonging to T, the segmentation results are recomposed to compute the final ki67-index estimate for T as the percentage of all the ki67 positive nuclei detected in T (i.e. the sum of the positive nuclei in all sub-images extracted from T), with respect to all the nuclei detected in T.

### Experimental results

After running the proposed prototype on our image database, three experts visually evaluated the segmentation results (E5, E15, E30), and we calculated the correlation between the computed ki67-index and their ki67-index estimates. The experts have, respectively, five, fifteen, and thirty years of experience in the field.

To test the developed system we firstly asked the three experts E5. E15, E30 to visually evaluate the nuclei detected by in all the 105 fields included in our study. All the three experts judged the results effective and trustable and considered them promising.

Secondly, for all the tumor areas in the 105 fields, we asked them to provide their ki67-index estimate, expressed as the percentage of nuclei positive for pki67 over all the nuclei (in the following, the estimates provided by E5, E15, E30 will be referred to as, respectively GT5, GT15, GT30).

With our prototype, we could provide three different estimates of the ki67-index:

1. **AreaEst**: an estimation of the ki67-index computed as the ratio of the area of the segmented positive nuclei to the area of all the segmented tumor nuclei. This estimation is motivated by the results presented in [24]. In their work, authors showed that the true ki67-index in tumor areas is approximated with a linear model of the area of positive to the total area of tumor nuclei.
2. **NoEst**: an estimation of the ki67-index computed by using the estimates of the number of the positive and the negative tumor nuclei. These estimates are computed by dividing the area of the positive nuclei by the estimated median area of the positive nuclei (medAP, see subsection 2.1), and the area of the negative nuclei by the estimated median area of the negative nuclei (medAN, see subsection 2.1).
3. **indexEst**: the estimation of the ki67-index computed as the percentage of detected nuclei positive to pki67 with respect to all the detected nuclei.

Given the experts’ estimates (GT5, GT15, GT 30), to measure the “agreement” with the automatically estimated estimates, we used the Pearson Correlation Coefficient (PCC).

Table 1 reports PCC among all the estimates provided by experts (GT5, GT15, GT30), and the measures (AreaEst, NoEst, and indexEst) computed for all the 105 fields.

**Table 1:** Pearson Correlation Coefficient between estimated ki67-index over all database

PCC	GT30	GT15	G5	EstArea_All_	EstNo_All_	indexEst_All_
GT30	1.00	0.89	0.76	0.83	0.87	0.94
GT15	0.89	1.00	0.80	0.85	0.86	0.87
GT5	0.76	0.80	1.00	0.69	0.68	0.73

Observing the first row of Table 1, it can be noted that the PCC between indexEst_All_ and GT30 (the most practiced expert) is higher than the PCC between GT30 and GT15, and GT30 and GT5, while the other two estimates (EastArea_All_ and EstNo_All_) obtain results PCCs comparable to those between GT15 and GT30.

Recalling that correlation between GT30 and GT15 was 0.89 and that correlation between GT30 and GT5 was 0.76, we may believe that the estimation algorithm, on all the database, performs as well trained clinical expert.

As detailed in section 5.1, our dataset contains 65 fields, acquired in different times, scanned with a resolution of 0.5 μm (20x), which will be referred to as DB20x in the following, and 40 fields, acquired in different times, scanned at a resolution 0.25 μm (40x), which will be referred to as DB40x in the following. To better investigate the algorithm performance with respect to the images resolution, we compared the results achieved by the algorithm, when applied separately on DB20x and on DB40x.

Table 2 shows the and PCC between GT30 and the estimates computed over the two databases (AreaEst_DB20x_, NoEst_DB20x_, IndexEst_DB20x_, AreaEst_DB40x_, NoEst_DB40x_, IndexEst_DB40x_, where the subscript shows the dataset where the measurements where estimated).

**Table 2:** Pearson Correlation Coefficient between estimated ki67-index

PCC	GT30	EstArea_DB20x_	EstNo_DB20x_	indexEst_DB20x_	EstArea_DB40x_	EstNo_DB40x_	indexEst_DB40x_
GT30	1.00	0.88	0.89	0.97	0.81	0.85	0.92
GT15	0.89	0.87	0.88	0.90	0.83	0.85	0.85
GT5	0.76	0.71	0.72	0.75	0.68	0.65	0.70

Observing Table 2, it becomes clear that the algorithm works much better when the resolution is lower. Indeed on DB20x, the performance increase with respect to those computed on the whole dataset, while performance computed on DB40x are worst (performance on all the database are obviously a balanced trade-off between those obtained on the separated database).

To better understand the cause of an error increase when the resolution is higher, we firstly visually analyzed images scanned at 40x resolution and we compared the results obtained at 20x resolution. Our observation highlighted that, when images containing nuclei agglomerates or when noise or color deposits are present, the algorithm processing images at 40x produces an higher number of over-segmentations than the algorithm processing the same images at 20x.

**Fig. 6:**
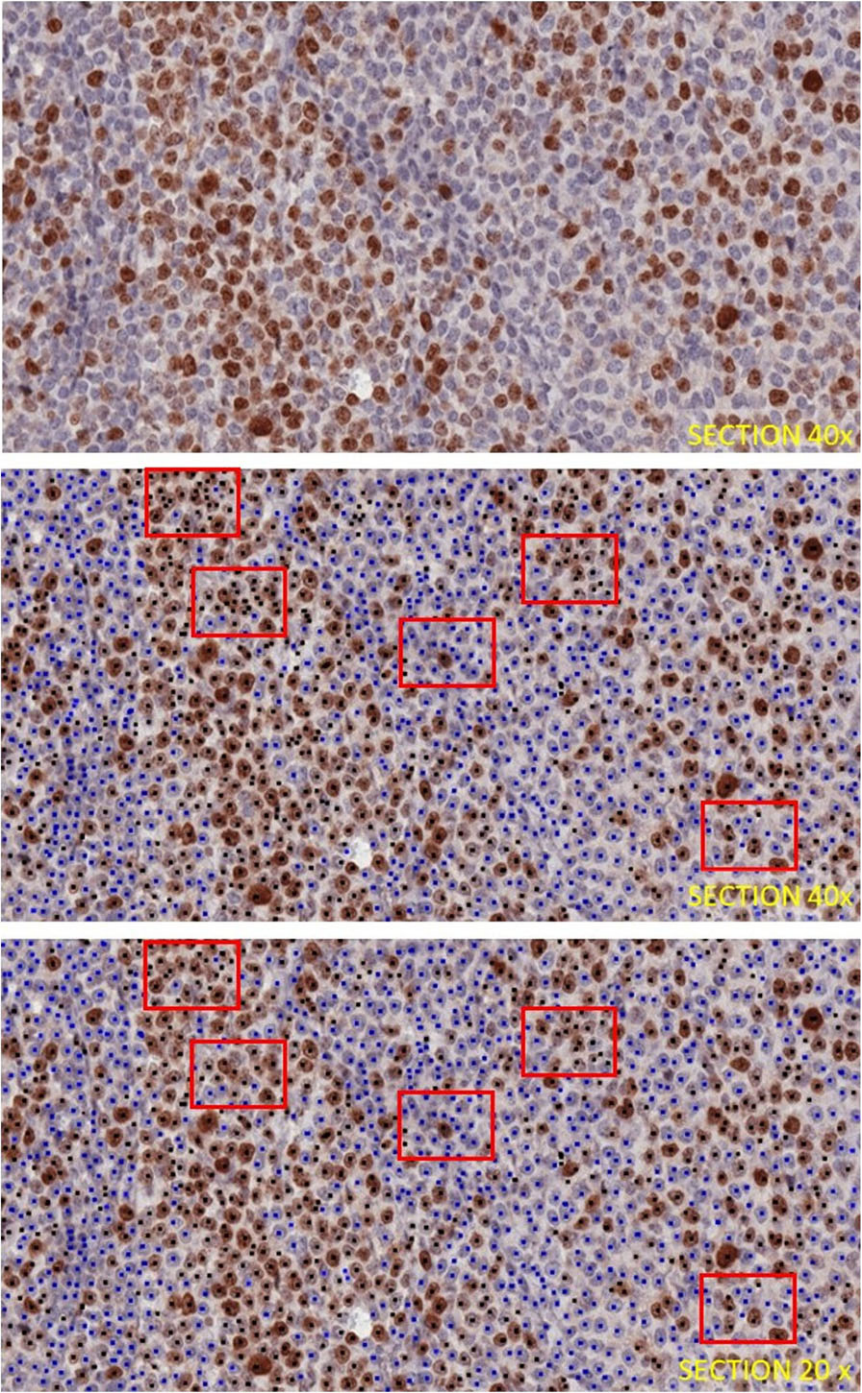
(Top) An image patch extracted from the tumor area of a tissue scanned at resolution 40x. (Center) the segmentation results show that over-segmentations occurred. (Bottom) when the image is downscaled to half its size, thus obtaining a 20x resolution, over-segmentation errors are resolved.

As an example, in the top of Fig. 6 we show a sub-image, whose original size is 2048 × 1024 pixels, which has been extracted from a section scanned at 40x. In the central row of the Fig. 6 the result produced by our algorithm is shown. Red rectangles highlight areas where over-segmentation, sometimes due to unspecific colorations or deposit, has occurred.

To effectively check that the high resolution increases the over-segmentation rate, we downscaled all the 40 fields in the DB40x database, we then reprocessed the downscaled images, we recomputed the correlations, and we also visually analyzed the achieved results. As expected, the amount of over-segmentation was drastically reduced and correlation with GT30 numerically showed it (the PCC between GT30 and EastArea increased to 0.85, the PCC between GT30 and EstNo increased to 0.88, and PCC between GT30 and IndexEst went up to 0.95).

In the bottom of Fig. 6 we show the segmentation results after such downsampling. Note that, in the red rectangles, there are no over-segmented nuclei.

To explain this over-segmentation effect, we note that the better performances on DB20x are surely depending on the fact that noise is more evident in high-resolution images and can therefore have more impact on the segmentation results; however, we believe that results are also influenced by the training set provided by experts. To explain this consideration, we recall that the training set consists of few manually signed nuclei positive for ki67 and few manually signed nuclei negative for ki67. The training set is used to train Bayesian trees that recognize the nuclei appearance and the nuclei shape. When experts sign training nuclei with very small areas, the algorithm tend to produce over-segmentations. Sections scanned at 40x resolution tend to clearly show also small areas, which are then signed by experts. When using sections scanned at 20x, the same areas appear less evident and are generally neglected by experts. We further recall that tissue sections are obtained by sectioning a 3D tissue volume, thus resulting in a 3D sectioning of cells themselves. Cells with a very light appearance and a spiculated shape (very similar to that of noise) in the obtained image sections are those that have been sectioned at the top or at the bottom of their height. The decision to include these cells into the count is left to experts, which either include or exclude them from the training set. We noted that experts tend to consider light cells when using 40x resolution, while they tend to neglect them when resolution is 20x. When experts train the system in order to detect light colored cells, the system becomes more sensitive to unspecific colorations due to color deposit or pigments, and may produce over-segmentations.

Anyway, it has to be noted that all the three estimates have correlations with GT30 which are comparable to that of the clinical expert with 15 years of experience. Moreover, though indexEst is the estimate that best correlates with experts, both the approximate methods described at the beginning of this section seem to produce estimates (AreaEst, NumberEst) that align well with the mean of the three experts. This fact somehow seems to confirm the results described in [24].

To understand if all the procedures composing our algorithm are necessary and to test the robustness with respect to the parameter settings, we performed tests by removing one procedure each time. Precisely, we removed the following steps by our method:

- preprocessing described in section 5.1.1 (obtaining estimates called **NoPreproc**); removing this step means that the parameters *N*, *M*, and *R* are set to *N* = 1, *M* = 1, *R* = 1, thus allowing us to test the extreme case.
- Log-based multiscale procedure described in subsection 2.5 (obtaining estimates called **NoLog**); removing this step means setting the parameter *perc*_*LOG*_ = 0, and performing no iterations.
- STRESS-based multiscale procedure described in subsection 2.6 (obtaining estimates called **NoStress**); removing this step means setting the parameter *perc*_*STRESS*_ = 0, and performing no iterations.

The estimates thus computed have been compared to GT30 and the estimates computed by our automatic algorithm over all the database Results, reported in Table 3.

**Table 3:** Comparison of estimates obtained by removing one of the main steps of the algorithm

PCC RMSE	GT30	indexEst	NoPreproc	NoLog	NoSTRESS
GT30	1.00	0.94	0.87	0.88	0.88
indexEst	0.94	1.00	0.93	0.95	0.96

The visual observation of the achieved results shows that the performance decrease when removing STRESS preprocessing is due to the fact that many marker pixels are not segmented so that entire marker areas are lost. Contemporaneously, removing one of the two multiscale iterative procedures cause nuclei clusters to be considered as a one big cell, thus causing under-segmentations.

Next, we varied the STRESS parameters to the extreme case where *N* = *maxint*, *M* = *maxint*, *R* = *Inf*, where *maxint* is the maximum integer value. In this case, the whole image is used to perform *maxint* iterations, and for each iteration, *maxint* samples are collected in the whole image. These parameters allow to obtain optimal enhancement results, but they increase the computational time of the algorithm, while the global performance does not increase (the PCC between GT30 and indexEst remains equal to 0.94), meaning that the chosen parameter settings are optimal.

## Discussion

In this paper we have presented a ki67-nuclei segmentation algorithm which produces effective results. Unfortunately, since publicly available and validated databases with labeled training images are not existing yet, the system performance could not be fairly compared to state of the art methods. Based on this consideration, we underline the urgent need of building such database.

In our future works we aim at:

1. extending the prototype to optimize its code and reduce its computational time;
2. developing a preprocessing step to analyze tissue sections and identify the cancerous tissue areas, where the ki67-index might be estimated with the proposed counting method;
3. developing a classification step to recognize tumor nuclei in the cancerous areas identified by the previous step;
4. integrating the developed prototype with the already developed MIAQuant software [47, 48].

## Conclusions

The pki67 is a marker of tumor aggressiveness [6, 7], and several research studies have already investigated the utility of its quantification in the prognostic and predictive evaluation of several types of tumors, such as breast, meningioma, soft tissue, lung, prostate, cervix and central nervous system cancers [8–17] and [18–21]. The expression of the pki67 in cancerous tissue areas is numerically quantified by the so-called ki67-index that is the percentage of tumor nuclei positive for pki67 over all the tumor nuclei. Given the high image resolution and dimensions, its estimation by expert clinicians is particularly laborious and time consuming. In this paper, we have presented a novel automatic approach for the estimations of the ki67-index, which needs only a limited number of training samples, that is nuclei manually signed experts. The presented approach starts by exploiting the STRESS algorithm [57] to produce an image enhancement (see Fig. 1) that allows to identify all the nuclei-pixels in the image by simply thresholding the “Stressed” image. Nuclei pixels are then input to a binary tree that classifies them as positive or negative to pki67 (see Figs. 1 and 2). To detach nuclei the algorithm exploits two multiscale procedures: the first applies LoG filters of different sizes, while the second employs “masked” versions of STRESS with differing radiuses. The nuclei detected by the two multiscale procedures are selected, or discarded, by a Bayesian tree recognizing eligible nuclei shapes. This procedure effectively identifies the nuclei (see Figs. 5 and 6). After processing both masks, the system computes the estimate of the ki67-index (indexEst) as the percentage of detected positive nuclei with respect to all the detected positive nuclei, and two rough ki67-index estimates (AreaEst and NumberEst).

The computed results have been evaluated both through three experts’ visual assessments and through the comparison of the computed indexes with those provided by the three experts (Table 1, Table 3). Though the method tend to produce over-segmentations when experts insert too small areas in the training set (see Fig. 6), both the aforementioned evaluations proved that the prototype is promising, so that experts believe in its potential as a tool to be exploited in the clinical practice as a valid aid for clinicians estimating the ki67-index.

## Methods

The software is implemented in MATLAB R2018; it is highly parameterized, it is easily extensible and modifiable to different users’ needs. Its source code is open source for any research purpose^1^.

### Image datasets

Our algorithm has been developed and tested on histological images of tumor specimens from subcutaneously xenotransplanted human lymphoma cells (SUDHL4) into female Severe Combined Immuno Deficiency (SCID) mice. The specimens were collected in the context of previous studies [63] performed at the “Fondazione IRCCS Istituto Nazionale dei Tumori” (Milan, Italy) in the framework of the project No. 9998 funded by Associazione Italiana per la Ricerca sul Cancro (AIRC) Special Program Molecular Clinical Oncology 5 per mille 2010 and approved by C.E.S.A. (Ethical Committee for Animal Experimentation, of the National Cancer Institute Foundation – see Additional file 1) and the Italian Ministry of Health [63].

Sections were stained for ki67 after antigen retrieval performed by heating in a pressure cooker with EDTA, 1 mM for 15 min. An UltraVision Quanto Detection System HRP (Thermo Fisher Scientific Inc.) and DAB (Liquid DAB + Substrate Chromogen System; Dako) were used to develop the reaction. Sections were scanned in differing times by using the Aperio ScanScope XT systems (Aperio Technologies, Leica Microsystems). Overall, the database currently contains 105 sections (fields): 65 sections/fields were scanned with a resolution of 0.5 μm (20x), while 40 fields were scanned at a resolution of 0.25 μm (40x). The 65 20x fields (referred to as DB20x in section 2.7) were acquired in: February 2019 (15 sections), May 2019 (30 sections) and July 2019 (20 sections). The 40 40x fields (referred to as DB40x in section 2.7) were acquired in: May 2019 (20 sections) and in July 2019 (20 sections). The resulting 20x image fields have an approximate dimension in the range [8000 × 8000, 25000 × 25000] pixels, while the 40x fields have an approximate dimension in the range [15000 × 17000, 45000 × 55000] pixels. Unfortunately, during each acquisition, the biological procedure used to stain the images was different. Therefore, a high color and noise variability characterizes the processed field dataset. Each tissue image represents an area of about [4 mm – 12.5 mm], where the tissue occupies a small portion of the image in a light background.

#### Image preprocessing

**Fig. 7:**
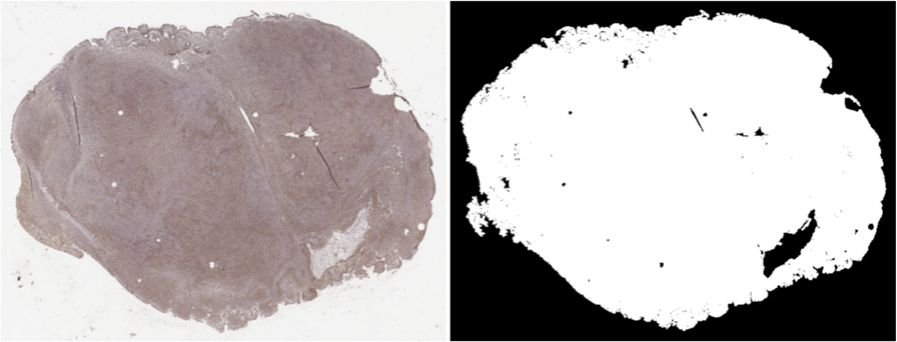
Tissue area segmentation results. Left: original tissue image. Right: segmentation mask

The described prototype has been developed with MATLAB R2018a, mainly using functions from the Statistics and Machine Learning Toolbox, and from the Image Processing Toolbox. To decrease computational load in terms of execution time and memory storage, we initially analyzed each image by applying the tissue-area segmentation procedure described in [47, 48]. The tissue area segmentation method is particularly efficient, and it effectively segments the tissue region allowing us to identify and discard both the background area and tissue holes or cuts. Figure 7 shows one of the processed tissue sections (left) and the segmented tissue area (right).

After identifying the tissue-area in each section, the next step is the (manual) identification of the tumor areas where the ki67-index must be estimated. To this aim, some automatic method have been presented at the state of the art, among which we recall the interesting work described in [24], where a deep convolutional neural network is used to recognize tumor areas. This method is interesting because the usage of a transfer learning technique reduces the number of samples needed for training. However, experiments exploiting it on our database obtained poor results and a high misclassification error, probably due to the lack of a training set with sufficient high cardinality. Besides, clinician often prefer to choose areas of interest depending on the clinical problem to be investigated. Therefore, in our work, tumor region identification is still manually performed, though we aim to develop an automatic algorithm in our future works. Overall, each field in our dataset contains 1 to 3 manually identified tumor regions of interest, resulting in 63 tumor regions in DB40x and 91 tumor regions in DB20x. Each tumor region is characterized by its own ki67-index estimate, which describes the proliferation activity of that tumor region.

After tumor areas are extracted, they are filtered to remove salt-and-pepper noise, as well as gaussian noise. To this aim, for the 20x images, we apply a median filter with a 3 pixel size and a gaussian filter with standard deviation σ = 0.5. Note that, since the method has been developed (and tuned) on 20x images, the parameter values must be changed when working on images with different resolutions. We simply decided to adapt all the parameters of the described method by multiplying their value according to the ratio between the new resolution and the 20x resolution. As an example, when 40x images are treated, all the parameters must be doubled $\left(\frac{40x}{20x}=\frac{1/0.25\upmu \mathrm{m}}{1/0.5\upmu \mathrm{m}}\right)$. Therefore, for 40x images, we use a median filter with a 7 pixel size (the median filter must have an odd size) and a gaussian filter with standard deviation σ = 1. This strategy is used to adapt the values of all the parameters in our method.

Next, each filtered tumor area is split into overlapping sub-images with a dimension of 512 × 512 pixels (the sub-image overlap is of 30 pixels). The splitting is applied to allow the parallel processing of each sub-image, to speed computation. After processing each sub-image, results are recomposed to obtain the final counts and estimate the ki67-index for the tumor region (as described at the end of section 2.6).

Anyway, after extracting patches from the 20x images, the obtained sub-image database contains about 50,000 images. After extracting patches from the 40x images, the obtained sub-image database contains about 110,000 images. For developing the prototype, we employed only 50 sub-images extracted from different tumor areas in the 15 fields of DB20x acquired in February. The 50 sub-images have been randomly chosen. All the remaining patches from DB20x and DB40x have been used for evaluating the prototype results. Figure 7 shows one of the processed tissue sections used for developing the method (left) and the segmented tissue area (right).

**Fig. 8:**
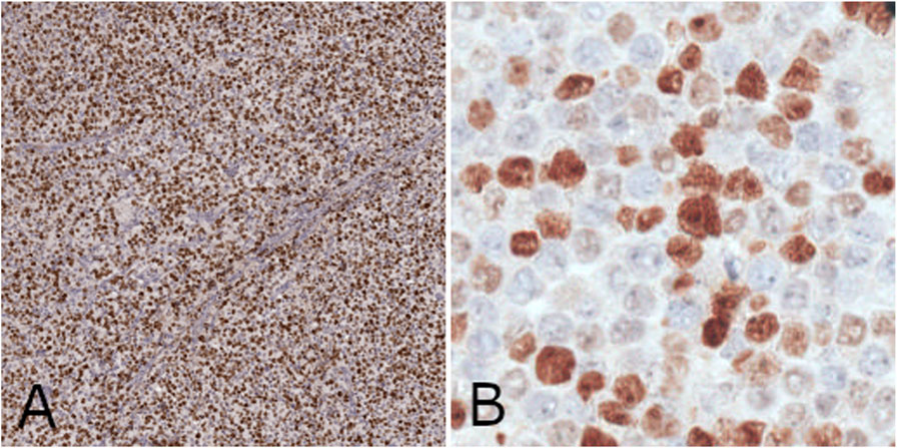
Left: example of the extracted sub-images with dimension 512x512 pixels. Nuclei positive for pki67 are the brown ones, while non-replicating cells are the light blue ones. Right: a detail of another sub-image.

Figure 8 shows (on the left) one of the sub-images extracted from the section in Fig. 7, and a zoomed detail of a sub-image extracted from another section image in our database. Observing the two sample images, it is apparent that the two sub-images are characterized by different color intensities, and that nuclei are often characterized by feeble color and low contrast. For this reason, they often appear as “shadows” and are difficult be detected.

## Additional file

**Additional file 1.** Declaration for Animal Experimentation.

## Data Availability

The data are not publicly available for they are still under usage by colleagues, did not approve their publication. Code is available from the corresponding author upon reasonable request.

## Abbreviations

ACE: Automatic color equalization
CAD: Computer aided diagnosis
DT: Bayesian decision tree
E15: Expert with 15 years of experience
E30: Expert with 30 years of experience
GT15: Ground truth estimations provided by E15
GT30: Ground truth estimations provided by E30
HDR: High dynamic range
HVS: Human visual system
IHC: Immunohistochemistry/immunohistochemical
LoG: Laplacian of Gaussian
PCC: Pearson correlation coefficient
pki67: protein ki67
SCA: Spatial color algorithm
SCID: Severe combined immuno deficiency
STRESS: Spatio-temporal retinex inspired envelope with stochastic sampling

## Footnotes

1 To get the zipped code, please write to elena.casiraghi@unimi.it.
