# Supplementary material for: ki67 nuclei detection and ki67-index estimation: a novel automatic approach based on human vision modeling
Source: BMC Bioinformatics. 2019 Dec 27;20:733. doi: 10.1186/s12859-019-3285-4 (PMC6935242; doi:10.1186/s12859-019-3285-4)
Supplement: Supplementary file 1 — Additional file 1. Declaration for Animal Experimentation. [file 12859_2019_3285_MOESM1_ESM.pdf]

FONDAZIONE IRCCS
ISTITUTO NAZIONALE
DEI TUMORI

Milan, February 8 2010

Declaration for Animal Experimentation

The C.E.S.A. (Ethical Committee for Animal Experimentation, of the National Cancer Institure Foundation) has evaluated the proposal.

The Committee considers that the preliminary results, obtained in in-vitro experiments deserve a preclinical study in vivo.

The procedures related to animal use are accurately described in the proposal and conform to all regulations protecting animals used for research purposes, including those of the DL 116/92. According to such law, every three years all the projects using laboratory animals are sent to Ministero della Salute for approval. The experiments described in the proposal will be performed following the detailed guidelines drawn-up by C.E.S.A. according to: Workamn P., et al. (1998) United Kingdom Coordinating Committee on Cancer Research (Guidelines for the welfare of animals in experimental neoplasia. Br.J.Cancer 77: 1-10).

Marco Alessandro Pierotti, PhD
President, Ethical Committee
for Animal Experimentation
